# Advanced Hierarchical Vesicular Carbon Co‐Doped with S, P, N for High‐Rate Sodium Storage

**DOI:** 10.1002/advs.201800241

**Published:** 2018-05-08

**Authors:** Guoqiang Zou, Hongshuai Hou, Christopher W. Foster, Craig E. Banks, Tianxiao Guo, Yunling Jiang, Yun Zhang, Xiaobo Ji

**Affiliations:** ^1^ State Key Laboratory for Powder Metallurgy Central South University Changsha 410083 China; ^2^ College of Chemistry and Chemical Engineering Central South University Changsha 410083 China; ^3^ Faculty of Science and Engineering Manchester Metropolitan University Chester Street M1 5GD Manchester UK; ^4^ College of Materials Science and Engineering Sichuan University Chengdu 611731 China

**Keywords:** electrochemistry, heteroatom doping, hierarchical vesicular carbon, sodium‐ion batteries

## Abstract

Hierarchical nanoscale carbons have received wide interest as electrode materials for energy storage and conversion due to their fast mass transfer processes, outstanding electronic conductivity, and high stability. Here, heteroatom (S, P, and N) doped hierarchical vesicular carbon (HHVC) materials with a high surface area up to 867.5 m^2^ g^−1^ are successfully prepared using a surface polymerization of hexachloro‐cyclotriphosphazene (HCCP) and 4,4′‐sulfonyldiphenol (BPS) on the ZIF‐8 polyhedrons. Significantly, it is the first time to achieve a controllability of the wall thickness for this unique carbon, ranging from 18 to 52 nm. When utilized as anodes for sodium ion batteries, these novel carbon materials exhibit a high specific capacity of 327.2 mAh g^−1^ at 100 mA g^−1^ after 100 cycles, which can be attributed to the expanded interlayer distance and enhanced conductivity derived from the doping of heteroatoms. Importantly, a high capacity of 142.6 mAh g^−1^ can be obtained even at a high current density of 5 A g^−1^, assigning to fast ion/electronic transmission processes stemming from the unique hierarchical vesicular structure. This work offers a new route for the fabrication/preparation of multi‐heteroatom doped hierarchical vesicular materials.

Carbon materials have realized a huge impact within the scientific community and have been extensively utilized in energy storage,^1^ catalysis,^2^ biosensors,^3^ and high‐flux membranes.^4^ Possessing useful physicochemical properties, carbon with various nanostructures have been received great devotions. Typically, carbon nanotubes (CNTs), explored by Dr. Iijima of Japan Electronics (NEC) in 1991, are composed of the single walled nanotubes (SWNT) and multiwalled nanotubes (MWNT). The CNTs exhibited giant activity in most fields of science and engineering by reason of their unprecedented physical and chemical properties.^5^ Additionally, many other carbon materials with a single nanostructure have also been developed, such as graphene,^6^ carbon nanosheets,^7^ hollow carbon nanospheres,^8^ etc. To further improve performances, hierarchical carbon materials are being explored.^9^ For instance, hierarchical carbons composed of nanowires/nanotubes (1D) with nanoparticles (0D) have been widely explored, utilized within catalysis, optoelectonics, and electronics.^10^ Furthermore, hierarchical nanocomposites composed of nanosheets (2D) and nanoparticles (0D)/nanowires (1D) have also been stated.^11^ A functionalized 3D hierarchical porous carbon has been prepared and delivered a high capacitance performance supercapacitor, showing that 3D nanoscale architecture can offer a fast electron pathway as well as shorten diffusion pathways for mass transfer. Although some processes have been achieved, carbon materials with new hierarchical nanostructure are urgently needed.

Carbon materials with hierarchical vesicular structure are very attractive in energy storage due to their fast mass transfer processes and multichannel electronic conductivity, exhibiting the merits of hollow carbon nanospheres and nanosheets. The current reports concerning a vesicular structure are mainly focused upon silicate materials due to their relatively controllable pore structure and surface properties.^12^ However, these vesicular mesoporous silicas/silicate materials deliver poor structural or hydrothermal stabilities. In 2004, a vesicular carbon was reported by Dai and co‐workers through using amphiphilic carbonaceous material and micelle as template.^13^ Moreover, an oxidative treatment process was employed to stabilize the as‐obtained structure. The vesicular carbon demonstrates a good structural stability at high temperatures. Until now, the preparation of the hierarchical vesicular carbon has rarely been reported within the literature. Latest studies have also indicated that surface functional groups, derived from doping of heteroatoms, play a significant role in enhancing the energy storage performances of the carbon materials.^14^ The doping of heteroatoms (N, O, B, S, or P) within the carbon materials can effectively modify the electronic conductivity, expand the interlayer spacing, and enhance the wettability of the active material.[[qv: 2,7a,14d,15]] For example, a sulfur‐doped disordered carbon has been prepared by Jiang et al. and displayed a high reversible capacity of 516 mA h g^−1^ at 0.02 A g^−1^.^16^ Therefore, multi‐heteroatoms doped hierarchical vesicular carbon material with enlarged interlayer spacing, enhanced electronic conductivity, large surface area, and rapid mass transfer process are highly desired for the SIBs. Tang et al. reported an one‐pot method to synthesize nanotubes through the polymerization reaction between hexachloro‐cyclotriphosphazene (HCCP) and 4,4′‐sulfonyldiphenol (BPS).^17^ Considering the existence of massive hydroxyl in BPS and N—H bonds/high absorbability of Zeolitic imidazolate framework‐8 (ZIF‐8) derived from its high surface area and porous structure, a new idea emerges in our hearts (**Scheme** **1**).

**Scheme 1:**
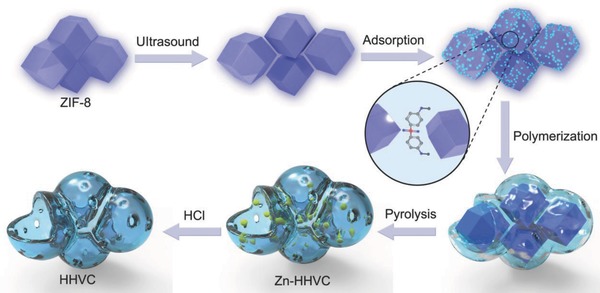
The schematic diagram of the preparation of heteroatoms doped hierarchical vesicular carbon (HHVC).

In this work, a series of multi‐heteroatoms doped hierarchical vesicular carbon (HHVC) materials are successfully prepared by through a surface polymerization of HCCP and BPS on the ZIF‐8. Impressively, the wall thickness of multi‐heteroatoms doped hierarchical vesicular carbon can be effectively controlled by the different amounts of HCCP and BPS. Furthermore, remarkable electrochemical storage performances for SIBs also were obtained with its unique structure. Additionally, the storage behaviors have been quantitatively assessed, approving a synergistic storage process of diffusion‐controlled intercalation behavior and surface‐induced capacitive behavior. This work provides a novel route for rationally designing multi‐heteroatoms doped hierarchical vesicular materials.

As shown in Figure S1a (Supporting Information), the ZIF‐8 presents a polyhedral shape, which agrees well with other reports.^18^ After a surface polymerization reaction of HCCP and BPS on ZIF‐8, the obtained intermediate products deliver an interconnected spherical structure, showing a smooth surface and an increased diameter of about 500 nm (Figure S1b,c, Supporting Information). As depicted in **Figure** **1**a–e, the HHVC‐150 exhibits a porous hierarchical vesicular structure, in which a large number of complete and incomplete vesicles can be clearly observed. Additionally, the diameter of the single vesicular is obviously less that of the intermediate products. Furthermore, the average wall thickness of the HHVC‐150 is about 18 nm. As displayed in Figure 1f, no long range ordered structure was observed in the HRTEM image of HHVC‐150, showing that the carbon is mainly amorphous. In the short range ordered region, the interlayer spacing is 0.41 nm, larger than the minimum spacing requirements (0.37 nm), benefiting the reversible insertion/extraction of the sodium ion.[[qv: 1a]] Energy‐dispersive spectroscopy (EDS) mappings indicate that the HHVC‐150 is composed of C, O, N, P, and S elements, in which these elements are homogenously distributed in the skeleton of the material (Figure 1g1–g5). The high dispersion status of heteroatoms proves that this design, to prepare the multi‐heteroatoms doped hierarchical vesicular carbon, is feasible and commendable.

**Figure 1:**
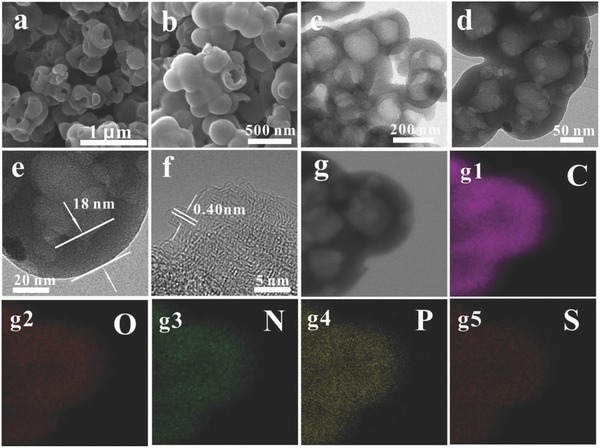
The SEM images (a,b), TEM images (c–e), the HRTEM image (f), and the EDS mapping (STEM, g–g5) of HHVC‐150.

As presented in **Figure** **2**a, the X‐ray diffraction (XRD) pattern of the HHVC‐150 shows two broad peaks located at 2θ degree of 22.2° and 41.6°, corresponding the (002) and (100) diffractions of carbon materials, which infers the amorphous structure of the obtained material.^19^ The interlayer distance for HHVC‐150 is 0.40 nm, calculating by the 2θ degree of (002) diffractions through the Bragg's equation, which is in good agreement with the HRTEM results. As shown in Figure 2b, the Raman spectrum delivers the two characteristic peaks at 1332.9 and 1604.3 cm^−1^, relating to the D and G bands of carbonaceous materials, which can be used to assess graphitization degree. The intensity ratio of *I*_D_/*I*_G_ is 0.917, indicating the existence of structural defects and graphitic structures.^20^

**Figure 2:**
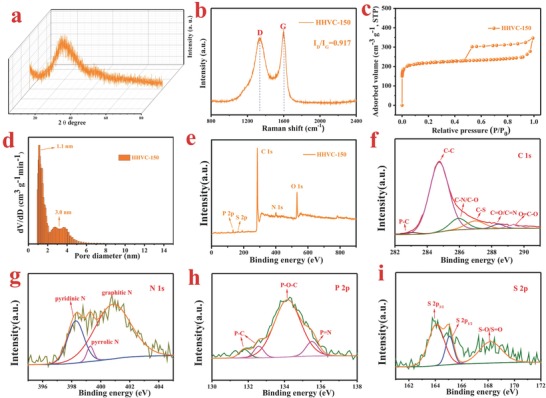
The XRD pattern (a), Raman spectrum (b), N_2_ adsorption/desorption isotherms (c), pore distribution (d), XPS survey (e), and high‐resolution XPS spectra of C1s (f), N1s (g), P2p (h), and S2p (i) of HHVC‐150.

Figure 2c displays the N_2_ adsorption–desorption isotherm of HHVC‐150, holding a typical IV isotherm shape with a hysteresis loop. The specific surface area of HHVC‐150 is 845.6 m^2^ g^−1^. The large surface area can provide more active sites for the storage of sodium ion through a fast capacitive process, rich channels for the mass transfer of sodium ion, and enhance the contact area between the active substance and the electrolyte, which can effectively modify the rate capabilities. Moreover, as presented in the Figure 2d, the pore diameter distribution of the sample is mainly located at 1.1 nm, showing the existence of micropores. Besides, there is small part of mesopores centered at 3.0 nm in the HHVC‐150.

XPS characterization was employed to measure the element contents and valence state in the materials. As depicted in Figure 2e and **Table** **1**, the XPS survey of the HHVC‐150 show the presence of the carbon, oxygen, nitrogen, sulfur, and phosphorus, corresponding element contents of 80.99%, 12.57%, 3.38%, 0.94%, and 2.12%, respectively. In the high resolution of the C1s spectrum (Figure 2f), the four peaks located at binding energies of 284.4, 285.3, 288.1, and 289.2 eV are ascribed to the C—C, C—O/C—N, C=O/C=N, and O—C=O bonds, separately.[[qv: 7b,21]] The two small peaks at 283.6 and 286.5 eV are assigned to the P—C and C—S bonds, respectively, suggesting that P and S atoms are implanted into the carbon lattice.[[qv: 15g,22]] In the high resolution of the N1s spectrum (Figure 2g), the three peaks centered at 398.8, 400, and 401 eV belong to the pyridinic‐N, pyrrolic‐N, and graphitic‐N, respectively.^23^ As described in Figure 2h, the high resolution of the P 2p spectrum presents four peaks located at 131.4, 132.7, 134.2, and 135.5 eV, which are related to the P—C, P—O—C, P—O, and P=N bond, respectively.[[qv: 15g,24]] As shown in Figure 2i, the deconvoluted spectrum of S 2p exhibits three peaks located at 164.0, 165.2, and 168.3 eV. The former two peaks can be attributed to S 2p_3/2_ and S 2p_1/2_ stemmed from the C—S bond.[[qv: 7b,25]] The peak located at 168.3 eV should be ascribed to the oxidized‐S (C—SO*_x_*—C).[[qv: 25a,26]] As shown above, it is powerfully demonstrates that the N, P, and S elements have been successfully introduced into the hierarchical vesicular carbon.

**Table 1:** The XPS composition analysis of the HHVC‐150, HHVC‐300, and HHVC‐600

Samples	Element content [%]				
	C	O	N	P	S
HHVC‐150	84.99	8.57	3.38	2.12	0.94
HHVC‐300	81.02	12.72	3.35	1.98	0.93
HHVC‐600	76.79	17.30	3.35	1.63	0.92

To further understand the impact of different amounts of HCCP and BPS on the morphologies and compositions of the HHVC, the HHVC‐300, and HHVC‐600 were also prepared (**Figure** **3**). As depicted in Figure 3b, the HHVC‐300 demonstrate a large number of complete vesicle with few incomplete vesicles, which is different with that in HHVC‐150 (Figure 3a). This phenomenon may be attributed to the increased wall thickness with the increasing HCCP and BPS. Note that the wall thickness of the HHVC‐300 is 34 nm. According to the scanning electron microscopy (SEM)/transmission electron microscopy (TEM) images of the HHVC‐600 listed in Figure 3c, the HHVC‐600 composes of numerous complete hollow spheres with an obviously increased diameter compared than that of the HHVC‐150 and HHVC‐300. Furthermore, the HHVC‐600 presents an interconnected hollow spherical structure with a thick shell of 52 nm, which may be attributed the thickening of the surface polymeric layer on ZIF‐8 with the increasing amounts of HCCP and BPS. These results indicate that the wall thickness and surface morphologies can be efficiently controlled by tuning the amounts of the HCCP and BPS, which is the first time to successfully realize the controllability of the thickness. Besides, as shown in Figure S2 (Supporting Information), the specific surface areas of the HHVC‐300 and HHVC‐600 are 564.5 and 348.6 m^2^ g^−1^. The decreased specific surface areas may be attributed to the increased wall thickness of the HHVC. More importantly, the ratios of the mesopores increase with the augment of the wall thickness. As shown in Figure S3 (Supporting Information) and Table 1, the contents of the carbon decrease from 84.99% to 76.79%, while the contents of oxygen change from 8.57% to 17.30% with the wall thickness range from 18 to 52 nm. Note that the contents of the N, P, and S are remaining unchanged. Additionally, as displayed in Figure S4 (Supporting Information) and Figure 2b, the values of the *I*_D_/*I*_G_ corresponding to the HHVC‐150, HHVC‐300, and HHVC‐600 are 0.917, 0.937, and 0.955, presenting a gradually increased trend, which may be attributed to the increase of oxygen contents.

**Figure 3:**
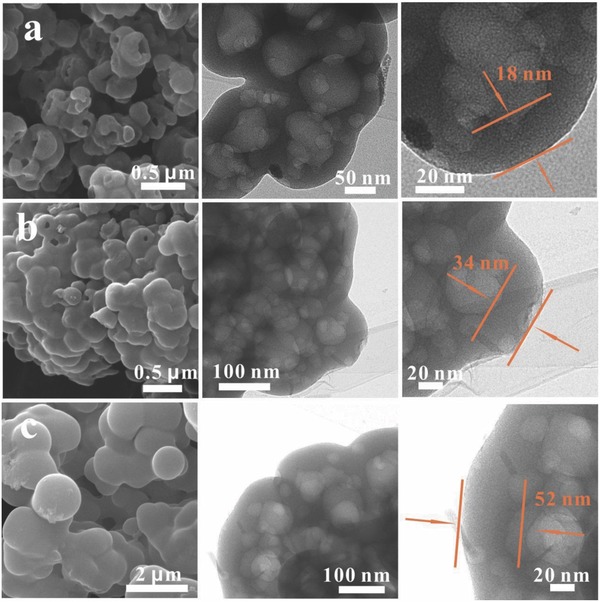
The SEM, TEM images of the HHVC‐150 (a), HHVC‐300 (b), and HHVC‐600 (c).

Taking all of these results together, a corresponding mechanism for the formation of HHVC is provided in Scheme 1. First, the ZIF‐8 was scattered to a certain extent by an ultrasound process for 5 min. Second, the BPS was adsorbed onto the surface of ZIF‐8 by the interaction of the hydrogen bonds. Third, surface polymeric layer was slowly formed after the addition of the excess trimethylamine (TEA). During the polymerization process, excess TEA can absorb the HCl to generate TEACl, which largely accelerates the polymerization reaction. Fourth, the as‐obtained products are pyrolyzed at 800 °C in Ar to form the Zn‐HHVC, in which the ZIF‐8 was broken down and acted as a N‐source. Finally, the HHVC can be obtained after a post acid treatment. Interestingly, the hierarchical vesicular structure of the material was kept well after the carbonization process.

The storage behavior of HHVC‐150 was tested via cyclic voltammetry (CV) by using Na‐half cell under a scan rate of 0.1 mV s^−1^ within the voltage range of 0.01 and 3 V. As presented in **Figure** **4**a, a large and broad cathodic peak range from 0.5 to 1.2 V was detected in the initial cycle, which may be assigned to the formation of the solid–electrolyte interphase (SEI) in the first cycle, some uncertain irreversible/side reactions between Na^+^ and surface defects/functional groups/electrolyte, and the decomposition of electrolyte (PC).[[qv: 15i,27]] The strong sharp cathodic peak centered at 0.01 V should be attributed to the insertion of sodium ion into the micropores/layers of the carbon. From the second to fifth cycles, a long cathodic slope without any overt bumps from 1.5 to 0.01 V and the small cathodic peak around at 0.8 V can be observed, owing to insertion/extraction of sodium ion into/out of the carbon layer with an expanded interlayer spacing (diffusion‐controlled process) or the reversible sodiation behavior in the surface functional groups, nanovoids and defects (capacitive‐controlled process), which happened in a wide voltage range.[[qv: 14c,28]] Besides, there is no obvious change observed in the CV curves after several cycles, indicating good cycling stability of HHVC‐150 materials

**Figure 4:**
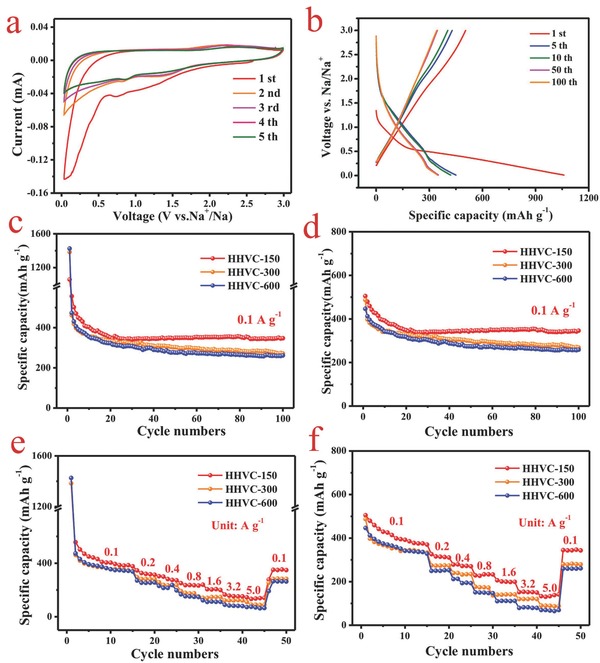
a,b) The CV curves at a sweep rate of 0.1 mV s^−1^ and galvanostatic discharge/charge profiles at 0.1 A g^−1^ of the SIBs utilizing the HHVC‐150 as anode, respectively. c,d) The galvanostatic discharge and charge tests of the obtained specimens under a current density of 0.1 A g^−1^ within the voltage range from 0.01 to 3 V (vs Na^+^/Na), separately. e,f) The rate performances of half‐cells employing HHVC as anodes.

Figure 4b displays the galvanostatic charge/discharge curves of HHVC‐150 under different cycles at 100 mA g^−1^ with a potential range between 0.01 and 3 V (vs Na^+^/Na). The first discharge/charge capacities of the HHVC‐150 electrode are 1060.2 and 504 mAh g^−1^ at a current density of 100 mA g^−1^. The capacity loss in the initial cycle may be attributed to the formation of the SEI layer or the irreversible/side reactions of the sodium ion, electrolyte, and the surface functional groups.[[qv: 19a]] Note that the slope in the low potential region (below 1.5 V) during the discharge process contributes most of the capacity, relating to insertion into the carbon layers or the reversible sodium storage through Na adsorption on the surface of carbon.

To measure the sodium storage performances of the HHVC, the HHVC‐300 and HHVC‐600 were also utilized as electrodes for SIBs. As depicted in Figure 4c,d, the HHVC‐150, HHVC‐300, and HHVC‐600 exhibit first discharge/charge capacities of 1060.2/504, 1384.3/487, and 1426.9/446.6 mAh g^−1^ at a current density of 100 mA g^−1^, corresponding first coulombic efficiencies of 47.6%, 35.2%, and 31.3%, respectively (Figure S5, Supporting Information). The relatively low first coulombic efficiencies of HHVC can be assigned to the irreversible reactions between organic electrolyte and active materials, and the formation of surface SEI film, which is equivalent to that of the other reports.[[qv: 14c,15i,29]] Interestingly, the first coulombic efficiencies are decreased from 47.6% to 31.3% with the increase of wall thickness, which may be related to some irreversible reactions between the sodium and surface oxygen‐containing functional groups (Table 1). After several cycles, the coulombic efficiencies of the HHVC materials can almost reach 100%. Furthermore, the HHVC‐150, HHVC‐300, and HHVC‐600 deliver reversible discharge capacities of 327.2, 271.7, and 259 mAh g^−1^ at a current density of 100 mA g^−1^ after 100 cycles. The gradual decrement in capacities of these materials may be attributed to changes in content of carbon and oxygen elements (Table 1; Figure S3, Supporting Information) and the different microstructures in surface area and pore distribution (Figure S2, Supporting Information).

High rate capability is a very important parameter for the practical application of these materials. As exhibited in Figure 4e and 4f, the HHVC‐150 presents an excellent rate performance with reversible capacities of 366.5, 314.4, 272.7, 233.6, 204.8, 151.9, and 142.6 mAh g^−1^ at current densities of 0.1, 0.2, 0.4, 0.8, 1.6, 3.2, and 5.0 A g^−1^, respectively. As a comparison, the HHVC‐300 and HHVC‐600 display specific capacities of 90.2 and 70.6 mAh g^−1^ under a high current density of 5.0 A g^−1^, obviously inferior than that of HHVC‐150. The good rate capability of the HHVC‐150 may be attributed to the fast mass transfer process benefited from the thin wall thickness, the hierarchical vesicular structure, and high surface area. Moreover, a capacity of 346.8 mAh g^−1^ can be obtained when the current density changes to 0.1 A g^−1^, suggesting a remarkable structural stability during the fast insertion/extraction processes. The comparison between HHVC and other carbon materials as anodes for SIBs reported in previous literatures is summarized in Table S1 (Supporting Information). The excellent electrochemical performances of the HHVC can be assigned to the heteroatoms‐doped hierarchical vesicular structure with a large interlayer spacing and high surface area, which can provide a fast transmission channel for electrons and sodium ions, and relieve the change in the volume.

Generally, the capacity sources of the carbon materials are stemmed from the insertion/extraction behavior of the Na^+^ into/out of the graphitic layer with a interlayer distance large than 0.37 nm and surface‐induced capacitive processes derived from the double‐layer capacitance and pseudocapacitance. To get a further insight into the storage behavior of the HHVC for SIBs, the relationship of the current density (*i*) and the scan rate (ν) can be utilized to measure the capacity source (Equation (1))

(1) $$i=av^{b}$$

where *a* and *b* are changeable indicators. *b* = 1 means that capacity contribution comes from surface‐controlled pseudocapacitive behavior, while *b* = 0.5 indicates a total diffusion‐controlled intercalation behavior.[[qv: 14d,28]] To obtain the *b* value, CV tests over an array of scan rates ranging from 0.1 to 5 mV s^−1^ of the HVVC samples were performed and the log(ν) − log(*i*_peak_) plots were fitted (**Figure** **5**a,d). The *b*‐value is the slope of the fitted line. As show in Figure 5d, the *b*‐values of the HHVC‐150 is 0.8, showing that the surface‐controlled pseudocapacitive behavior and diffusion‐controlled intercalation behavior contribute the capacity simultaneously. As a contrast, the *b*‐values of HHVC‐300 and HHVC‐600 are also provided. The *b*‐value of HHVC‐600 is 0.51, inferring that the total capacity stems mainly from the intercalated process, which may be mainly attributed to the relatively small specific surface area and thick wall thickness. These results suggest that the increased wall thickness is adverse to the fast surface capacitive behavior, and thus explains a relatively poor rate capability of HHVC‐600 than that of HHVC‐150, which is well accord with the experiment results. Furthermore, Equation (2) was used to quantify the capacitive contribution of HHVC‐150

(2) $$i(V)=k_{1}v+k_{2}v^{1/2}$$

where *k*_1_ν and *k*_2_ν^1/2^ are separately assigned to the contribution of surface capacitive behavior and intercalated behavior, in which *k*_1_ and *k*_2_ are related the slope and intercept of linear plots (ν^1/2^ − *i*(*V*)/ν^1/2^)(Figure S6, Supporting Information), respectively.^30^ As exhibited in Figure 5e, the capacitive contributions decrease from 68.4% to 56.4% when the potential increased from 0.5 to 1.5 V under a scan rate of 0.5 mV s^−1^, demonstrating that a relatively low voltage is favor of the occurrence of surface‐induced capacitive processes. Additionally, the capacity contributions of the HHVC‐150 under various sweep rates at 0.5 V are listed in Figure 5f. The capacitive contributions in the whole charge storage are increased from 46.3% to 85.9% when the sweep rates are changed from 0.1 to 5 mV s^−1^, illustrating that the capacity mainly originates from fast surface‐induced capacitive behavior under high current density since the sluggish intercalation speed cannot fulfil the needs of the fast electrochemical processes.

**Figure 5:**
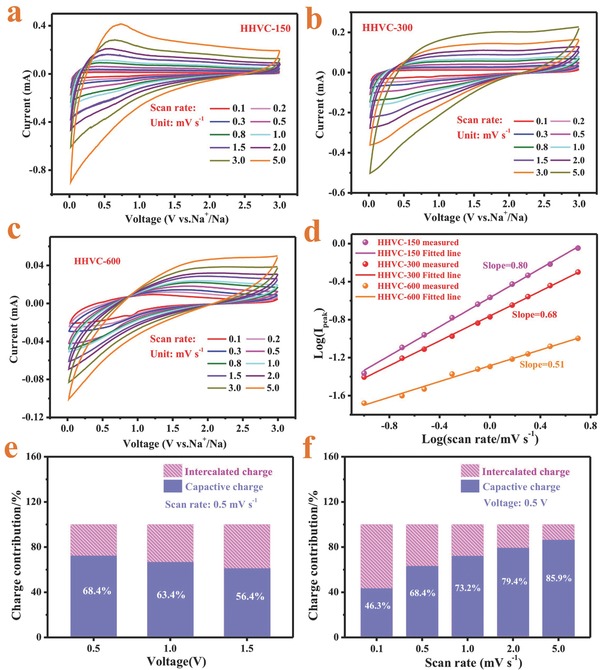
a–c) The CV curves measured under different scan rates range from 0.1 to 5 mV s^−1^ of HHVC‐150, HHVC‐300, and HHVC‐600, respectively. d) The correlation of the peak currents (*i*) and scan rates (ν) (log(sweep rate, mV s^−1^) − log(*I*_peak_)). e) Capacity contributions of capacitive and intercalated processes at 0.5, 1.0, and 1.5 V for HHVC‐150 at a scan rate of 0.5 mV s^−1^. f) Capacity contributions of capacitive and intercalated processes under the scan rates range from 0.1 to 5.0 mV s^−1^ for HHVC at a certain potential of 0.5 V.

In summary, heteroatoms (S, P, and N) doped hierarchical vesicular carbon materials with high surface areas and large interlayer spacing have been designed by a surface polymerization reaction. Besides, the wall thickness of HHVC was first manipulated by tuning the amounts of HCCP and BPS, delivering a wall thickness ranged from 18 to 54 nm. The related formation process of HHVC has also been provided. When these novel structures were employed as electrode for SIBs, the HHVC presented an excellent electrochemical storage performance. Furthermore, calculated results indicate that fast surface‐induced capacitive behavior contributes to most of the capacity during fast discharge/charge processes, confirming that the outstanding rate capability of the carbon materials can be realized by the fast capacitive behavior. Significantly, this paper describes a novel method to prepare hierarchical vesicular carbon materials, providing the foundation for the fabrication of other hierarchical vesicular materials.

## Experimental Section

*Materials Synthesis—Preparation of ZIF‐8 Polyhedrons*: Typically, 0.9 g of Zn(NO_3_)_2_·6H_2_O was dissolved in 20 mL of methanol, the obtained solution was denoted A. Then, 1.98 g 2‐methylimidazole was dissolved in 10 mL of deionized water, the prepared solution was marked as B. A was decanted into B under magnetic stirring. After vigorous stirring for 10 min at room temperature, the purple precipitate ZIF‐8 was obtained by washing thoroughly with methanol, and drying under vacuum at 80 °C overnight.

*Materials Synthesis—Preparation of HHVC*: 100 mg above‐prepared material was immersed into 40 mL methanol solution through an ultrasound process at room temperature for 20 min, the obtained mixture was flagged as C. Then, 150 mg HCCP and 335 mg BPS were dissolved in 20 mL methanol solution, the as‐obtained clear solution recorded as D. Subsequently, D was dropwise added into C by using a separating funnel. Excess TEA (1 mL) was joined slowly after vigorous stirring for twenty minutes. The reaction mixtures were vigorous stirred at room temperature for 12 h. The yielded product was filtrated and then washed three times with methanol. After drying at 60 °C for 10 h, the obtained samples was pyrolyzed at 900 °C for 1 h with a heating ramp of 3 °C min^−1^ under N_2_ atmosphere. Finally, the obtained black solid was treated by 40 mL 2 m HCl under 100 °C. After washing with deionized water to pH = 7, the product was dried in vacuum at 80 °C for 6 h. The as‐obtained carbon material was denoted as HHVC‐150. As comparison, the carbon materials prepared from 300 mg HCCP/670 mg BPS and 600 mg HCCP/1440 mg BPS were marked as HHVC‐300 and HHVC‐600, respectively.

*Materials Characterization*: XRD was measured on an X‐ray diffractometer (Rigaku) (*V* = 30 kV, *I* = 25 mA, 0.15418 nm). Raman spectra were performed at using a Raman spectroscopy (DXR, Thermo‐Fisher Scientific). SEM and TEM were tested on a Hitachi S‐4800 microscope and a JEOL‐2010 instrument, respectively. X‐ray photoelectron spectroscopy and nitrogen adsorption/desorption isotherms were separately collected by VG Multi Lab 2000 system and an ASAP 2010 instrument.

*Electrochemical Measurements*: Slurry composed of 70 mg active materials (HIVC‐150, HIVC‐300, and HIVC‐600), 15 mg binder CMC (carboxymethyl cellulose), 15 mg Super P (conductive carbon), and moderate deionized water was evenly dispersed on the Cu foils and dried at 90 °C in vacuum oven overnight. The average mass loading of the electrode is about 1.0–1.1 mg cm^−2^. Coin cells (CR2016‐type half‐cells) made up of the working electrodes, the counter, electrolyte, and separator were assembled in a glovebox (mBraun, Germany) with high purity argon atmosphere, in which the counter, electrolyte, and separator are the sodium metal, 1 mol L^−1^ NaClO_4_ solution in propylene carbonate, and polypropylene film, respectively. The galvanostatic charge/discharge tests at different current densities within a voltage window of 0.01–3 V (vs Na/Na^+^) and CV curves with different scan rates were collected on an Arbin BT2000 instrument and A Multi Autolab M204 electrochemical station, respectively.

## Conflict of Interest

The authors declare no conflict of interest.

## Supporting information

Supplementary
