# Supplementary material for: Advanced Hierarchical Vesicular Carbon Co‐Doped with S, P, N for High‐Rate Sodium Storage
Source: Adv Sci (Weinh). 2018 May 8;5(7):1800241. doi: 10.1002/advs.201800241 (PMC6051379; doi:10.1002/advs.201800241)
Supplement: Supplementary file 1 — Supplementary [file ADVS-5-1800241-s001.pdf]

Copyright WILEY-VCH Verlag GmbH & Co. KGaA, 69469 Weinheim, Germany, 2018.

Supporting Information

for *Adv. Sci.,* DOI: 10.1002/advs.201800241

Advanced Hierarchical Vesicular Carbon Co-Doped with S, P, N for High-Rate Sodium Storage

*Guoqiang Zou, Hongshuai Hou, Christopher W. Foster, Craig E. Banks, Tianxiao Guo, Yunling Jiang, Yun Zhang, and Xiaobo Ji**

Copyright WILEY-VCH Verlag GmbH & Co. KGaA, 69469 Weinheim, Germany, 2016.

Supporting Information

Title: Advanced Hierarchical Vesicular Carbon Co-doped with S, P, N for High-rate Sodium Storage

Guoqiang Zou[1,2], Hongshuai Hou[1,2], Christopher W. Foster[3], Craig E. Banks[3], Tianxiao Guo[1,2], Yunling Jiang[1,2], Yun Zhang[4], and Xiaobo Ji[1,2]*

[1] G. Zou, Dr. H. Hou, T. Guo, Y. Jiang, and Prof. X. Ji
State Key Laboratory for Powder Metallurgy, Central South University, Changsha 410083, China

[2] College of Chemistry and Chemical Engineering, Central South University, Changsha, 410083, China
E-mail: xji@csu.edu.cn

[3] Dr. Christopher W. Foster, and Prof. Craig E. Banks
Faculty of Science and Engineering, Manchester Metropolitan University, Chester Street, Manchester, M1 5GD

[4] Prof. Y. Zhang
College of Materials Science and Engineering, Sichuan University, Chengdu, 611731, China

Table S1 The comparison of HHVC and other carbon materials as anodes for SIBs reported in previous reports

**Carbon materials**	**Cycling performance ($mAh\ g^{-1}$)**	**Rate capability ($mAh\ g^{-1}$)**	**References**
Natural graphite	~100 at 0.5 A g^{-1} after 2500 cycles	~ 145 at 0.2 A g^{-1}<br>~ 112 at 3 A g^{-1}	1
N-doped carbon nanosheets	155 at 0.05 A g^{-1} after 200 cycles	~190 at 0.2 A g^{-1}<br>~ 50 at 2 A g^{-1}	2
N-Doped reduced graphene	~100 at 0.1 A g^{-1} after 1000 cycles	~ 52 at 2.4 A g^{-1}	3
Carbon microspheres	~183 at 30 mA g^{-1} after 50 cycles	~149 at 0.1 A g^{-1}<br>~80 at 1 A g^{-1}	4
Hollow carbon nanospheres	~160 at 0.1 A g^{-1} after 100 cycles	~142 at 0.5 A g^{-1}<br>~100 at 2 A g^{-1}	5
3D porous carbon frameworks	~303.2 at 0.1 A g^{-1} after 100 cycles;	~290 at 0.2 A g^{-1}<br>~104 at 10 A g^{-1}	6
S-covalently bonded graphene	150 at 1 A g^{-1} after 200 cycles	262 at 0.1 A g^{-1}<br>161 at 1 A g^{-1}<br>83 at 5 A g^{-1}	7
S-doped flexible graphene films	244 at 0.1 A g^{-1} after 300 cycles	377 at 0.1 A g^{-1}<br>89 at 1 A g^{-1}	8
S-doped carbon	302.2 at 0.5 A g^{-1} after 700 cycles	192.5 at 2 A g^{-1}<br>119.5 at 5 A g^{-1}	9
S/N-codoped hollow carbon spheres	180 at 0.5 A g^{-1} after 200 cycles;	250 at 0.1 A g^{-1}<br>140 at 5 A g^{-1}	10
Hard carbon microtubes	~305 at 30 m A g^{-1} after 100 cycles	~275 at 0.15 A g^{-1}<br>~180 at 0.3 A g^{-1}	11
P/N-codoped carbon	275 at 0.1 A g^{-1} after 90 cycles	305 at 0.1 A g^{-1}<br>189 at 1 A g^{-1}<br>136 at 5 A g^{-1}	12
HHVC	~327.2 at 0.1 A g^{-1} after 100 cycles	~ 272.7 at 0.4 A g^{-1}<br>~ 204.8 at 1.6 A g^{-1}<br>~ 142.6 at 5 A g^{-1}	This work

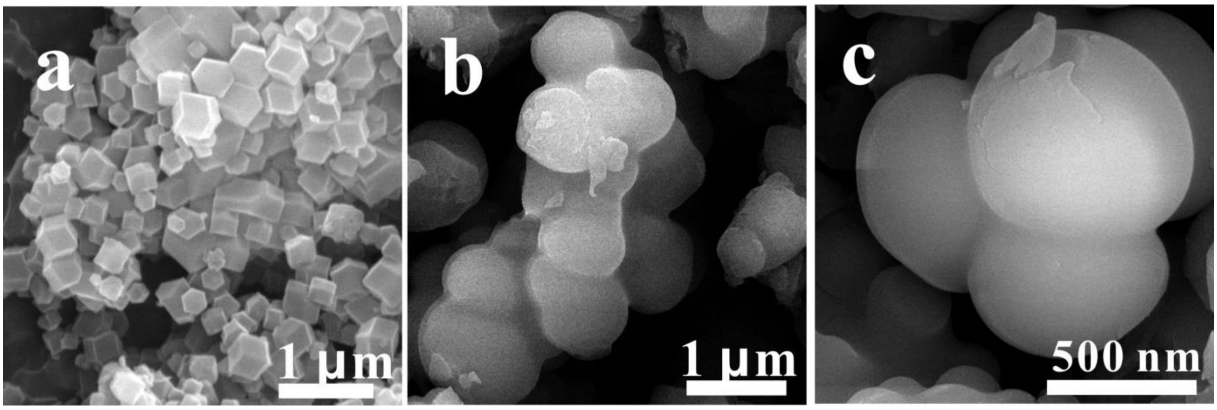

Figure S1 (a) The SEM image of ZIF-8, (b-c) the SEM images of the mid-product.

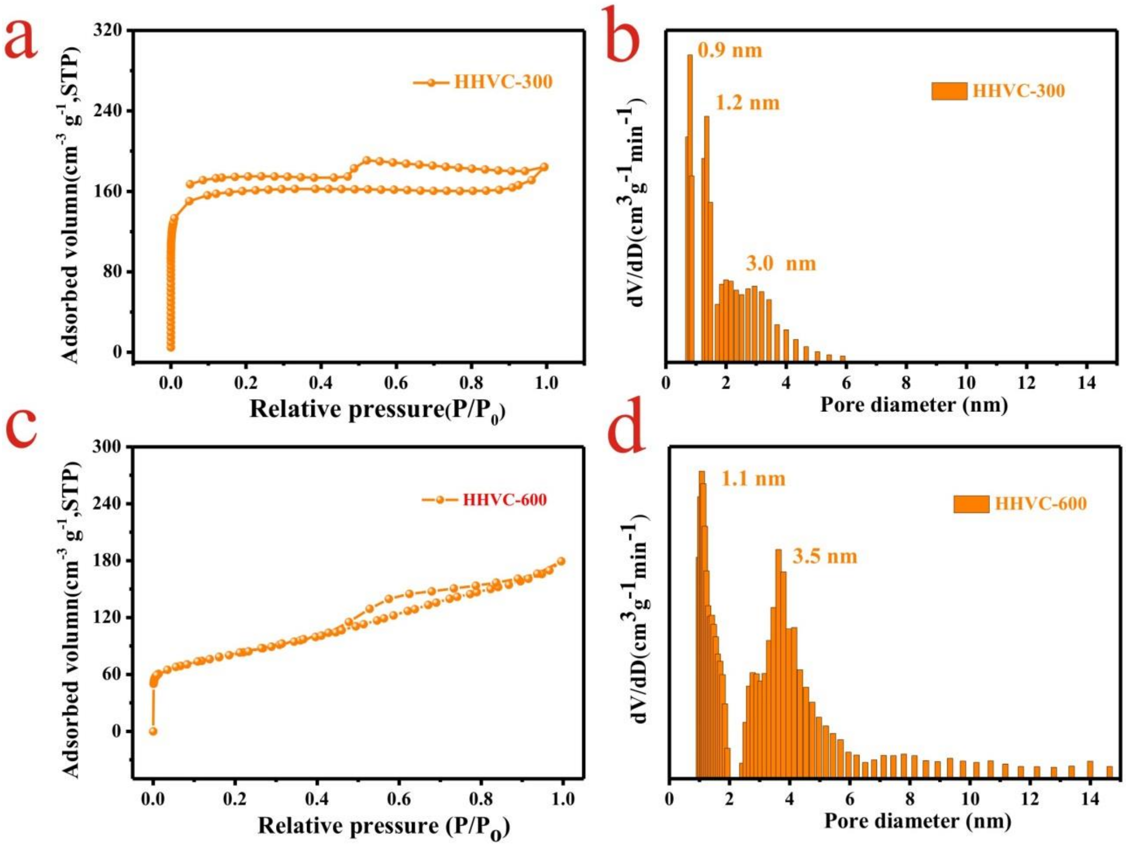

Figure S2 The N_2 absorption-desorption isotherms and pore diameter of the as-obtained samples.

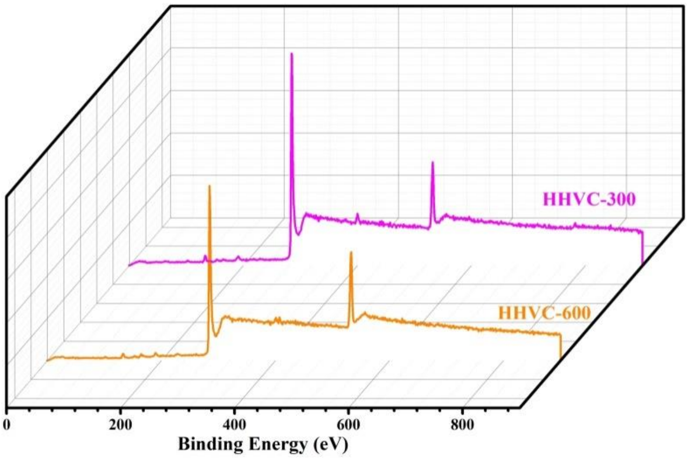

Figure S3 The XPS surveys of the HHVC-300 and HHVC-600.

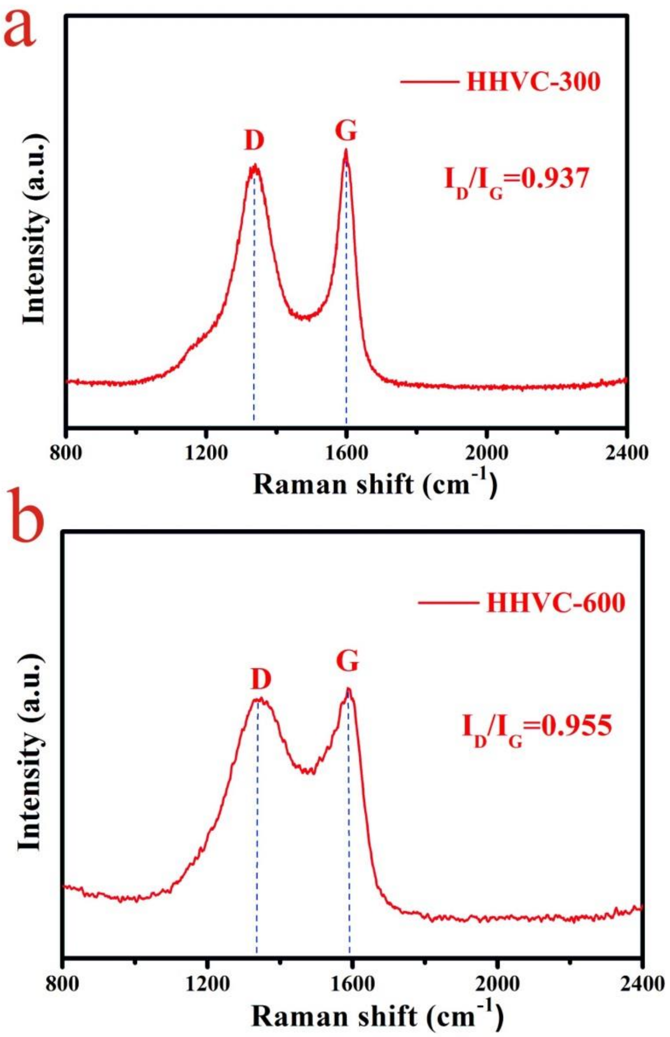

Figure S4 The Raman spectra of the HHVC-300 and HHVC-600.

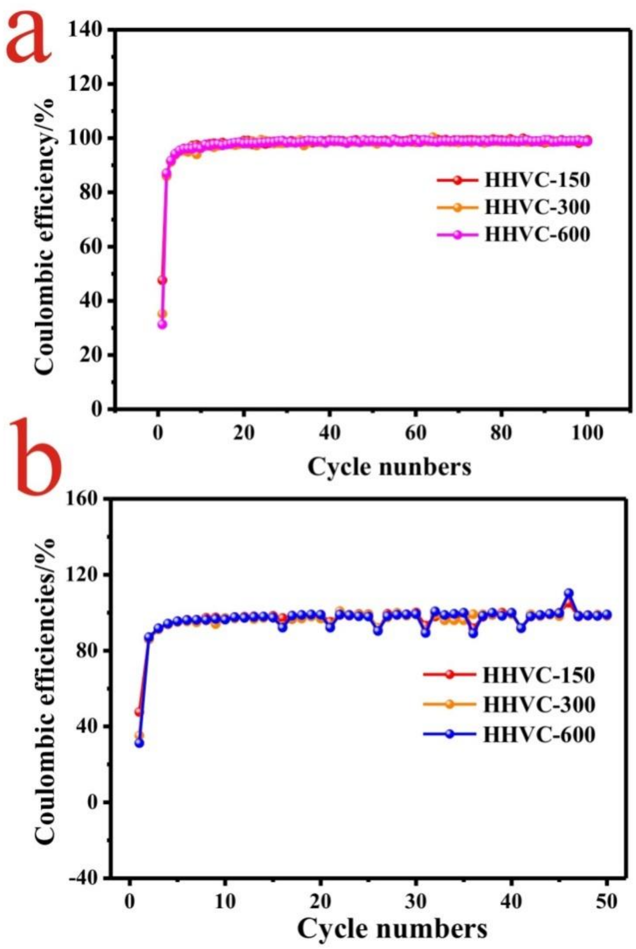

Figure S5 (a) The cycling coulombic efficiencies of the relating samples at 100 mAh g^{-1}, (b) the coulombic efficiencies of the relating samples under different current densities.

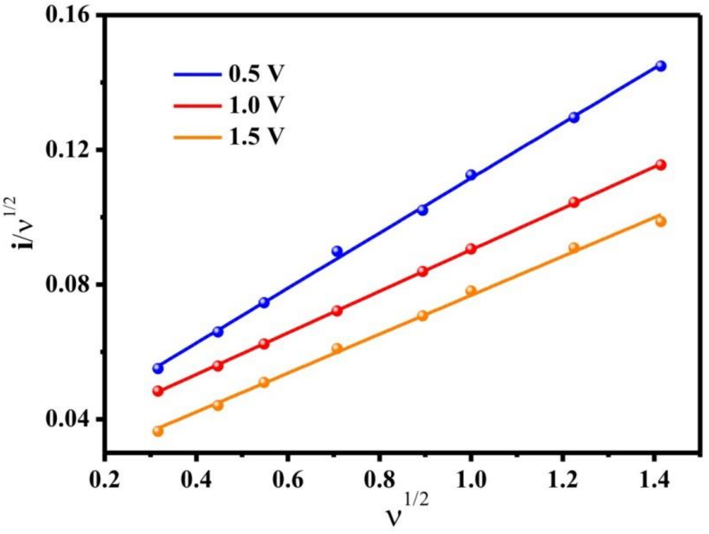

Figure S6 The fitted lines of $i/\nu^{1/2}$ vs $\nu^{1/2}$ at the voltages of 0.5, 1.0, and 1.5 V of SIBs using the HHVC-150 as anode.

References

1. Y. Liu, F. Fan, J. Wang, Y. Liu, H. Chen, K. L. Jungjohann, Y. Xu, Y. Zhu, D. Bigio and T. Zhu, Nano Lett., 2014, 14, 3445-3452.
2. H. G. Wang, Z. Wu, F. L. Meng, D. L. Ma, X. L. Huang, L. M. Wang and X. B. Zhang, Chemsuschem, 2013, 6, 56.
3. L. David and G. Singh, J. Phys. Chem. C, 2014, 118, 28401-28408.
4. T. Chen, L. Pan, T. Lu, C. Fu, D. H. C. Chua and Z. Sun, J. Mater. Chem. A, 2013, 2, 1263-1267.
5.K. Tang, L. Fu, R. J. White, L. Yu, M. M. Titirici, M. Antonietti and J. Maier, Adv. Energy Mater., 2012, 2, 873-877.
6. H. Hou, C. E. Banks, M. Jing, Y. Zhang and X. Ji, Adv. Mater., 2015, 27, 7861-7866.
7. X. Wang, G. Li, F. M. Hassan, J. Li, X. Fan, R. Batmaz, X. Xiao and Z. Chen, Nano Energy, 2015, 15, 746-754.
8. X. Deng, K. Xie, L. Li, W. Zhou, J. Sunarso and Z. Shao, Carbon, 2016, 107, 67-73.
9. L. Qie, W. Chen, X. Xiong, C. Hu, F. Zou, P. Hu and Y. Huang, Adv. Sci., 2015, 2, 1500195.
10. J. Ye, J. Zang, Z. Tian, M. Zheng and Q. F. Dong, J. Mater. Chem. A, 2016, 4, 13223-13227.
11. Y. Li, Y. S. Hu, M. M. Titirici, L. Chen and X. Huang, Adv. Energy Mater., 2016, 6, 1600659.
12. Y. Li, Z. Wang, L. Li, S. Peng, L. Zhang, M. Srinivasan and S. Ramakrishna, Carbon, 2016, 99, 556-563.
